# Neuropsychology, Autobiographical Memory, and Hippocampal Volume in “Younger” and “Older” Patients with Chronic Schizophrenia

**DOI:** 10.3389/fpsyt.2015.00053

**Published:** 2015-04-21

**Authors:** Christina Josefa Herold, Marc Montgomery Lässer, Lena Anna Schmid, Ulrich Seidl, Li Kong, Iven Fellhauer, Philipp Arthur Thomann, Marco Essig, Johannes Schröder

**Affiliations:** ^1^Section of Geriatric Psychiatry, Department of General Psychiatry, University of Heidelberg, Heidelberg, Germany; ^2^Center for Mental Health, Klinikum Stuttgart, Stuttgart, Germany; ^3^Department of General Psychiatry, Center of Psychosocial Medicine, University of Heidelberg, Heidelberg, Germany; ^4^German Cancer Research Center, Heidelberg, Germany; ^5^Institute of Gerontology, University of Heidelberg, Heidelberg, Germany

**Keywords:** chronic schizophrenia, autobiographical memory, hippocampus, structural magnetic resonance imaging, episodic memory, semantic memory, neuropsychology

## Abstract

Despite a wide range of studies on neuropsychology in schizophrenia, autobiographical memory (AM) has been scarcely investigated in these patients. Hence, less is known about AM in older patients and hippocampal contribution to autobiographical memories of varying remoteness. Therefore, we investigated hippocampal volume and AM along with important neuropsychological domains in patients with chronic schizophrenia and the respective relationships between these parameters. We compared 25 older patients with chronic schizophrenia to 23 younger patients and an older healthy control group (*N* = 21) with respect to AM, additional neuropsychological parameters, and hippocampal volume. Personal episodic and semantic memory was investigated using a semi-structured interview. Additional neuropsychological parameters were assessed by using a battery of standard neuropsychological tests. Structural magnetic resonance imaging data were analyzed with an automated region-of-interest procedure. While hippocampal volume reduction and neuropsychological impairment were more pronounced in the older than in the younger patients, both groups showed equivalent reduced AM performance for recent personal episodes. In the patient group, significant correlations between left hippocampal volume and recent autobiographical episodes as well as personal semantic memories arose. Verbal memory and working memory were significantly correlated with right hippocampal volume; executive functions, however, were associated with bilateral hippocampal volumes. These findings underline the complexity of AM and its impairments in the course of schizophrenia in comparison to rather progressive neuropsychological deficits and address the importance of hippocampal contribution.

## Introduction

Volumetric alterations in a variety of memory-associated cerebral regions such as the hippocampus (1, 2) as well as mnestic deficits have been frequently reported in schizophrenia (3–5). However, older patients with a chronic course of the disease have been scarcely investigated (6), although this patient group is expected to increase given demographic changes (7).

Hippocampal volume reductions of 2–4% were reported in schizophrenia (8, 9), and a significant volumetric reduction seems to be comparable in first-episode and chronic patients (2). Furthermore, a selective reduction of anterior (10–15) and posterior hippocampal volume (16–21) was repeatedly described in patients with schizophrenia. A regional specific pattern of hippocampal volume loss is of interest due to possible functional implications of hippocampal connections and cellular organization (22), as described in the hippocampal encoding/retrieval (HIPER) model (23): hippocampal activity (via positron emission tomography, PET) associated with memory encoding is primarily located in the anterior part; during memory retrieval, mainly, the posterior part is activated. Besides these findings, several studies reported specific relationships between hippocampal volume and cognitive abilities in schizophrenia (24, 25). Nevertheless, older patients with a duration of the disease of several decades were less frequently examined, with hippocampal volume reductions of 10–33% in relation to healthy comparison subjects (26–28).

Similarly, autobiographical memory (AM), which includes both personal episodic and semantic memory, was scarcely examined in patients with schizophrenia and studies focused primarily on younger patients (29–33). Our previous results from 33 older schizophrenic patients (mean age 52.03 ± 8.76) indicate consistently with these studies that poorer episodic and semantic AM performance of the patients is associated with left hippocampal volume reduction (34). However, differences in AM performance between older and younger patients with chronic schizophrenia are unknown as well as the relationships between AM from recent and remote lifetime periods and hippocampal volume. Therefore, we enlarged the patient sample described above and contrasted the respective results of elderly patients to that of an age-matched healthy control group and younger patients with a chronic course of the disease.

Concerning long-term memories – as assessed by autobiographical interviews – the contribution of hippocampus to recent and remote memories is discussed by two major theories making different predictions: The standard model of consolidation postulates a gradual process of reorganization of declarative memories, which become independent of medial temporal lobe structures as cortical regions become increasingly engaged (35–37). According to multiple-trace-theory, the hippocampus is permanently involved in remembering detailed and vivid episodic memories, while semantic memories become independent of this structure (38–40). Based on multiple-trace-theory, the transformation hypothesis proposes that during retrieval of vivid and detailed episodic memories, processes of re-encoding by the hippocampus allow the extraction of regularities. These semantic memories containing schematic and abstract information are represented by neocortical structures (41, 42).

In the present study, we used an automated region-of-interest analysis technique to examine hippocampal volume of older patients with chronic schizophrenia in contrast to younger patients and healthy controls. Given a functional segregation of anterior and posterior hippocampus, a differentiating analysis may help to identify the underlying pathological processes in schizophrenia and the associated cognitive implications. Considering different lifetime periods, we aimed to analyze AM performance of the respective groups and the specific associations between hippocampal volume and recent and remote episodic and semantic AM, respectively. A battery of standard neuropsychological tests was additionally applied, which require verbal memory, short-term and working memory, information processing speed, executive functions, and remote semantic memory to contrast with AM performance.

We hypothesized (1) hippocampal volume reductions and (2) AM as well as additional neuropsychological deficits especially in the older patient group in contrast to healthy controls. Furthermore, we expected (3) AM and (4) additional neuropsychological parameters of memory and executive functions to be correlated with hippocampal volume in both patients with schizophrenia and healthy controls.

## Materials and Methods

### Subjects

Twenty-five older (55.92 ± 6.85 years of age) and 23 younger patients (32.78 ± 7.00 years of age) with DSM-IV (43) schizophrenia or schizoaffective disorder were recruited from the residential care St. Thomas e.V., Heidelberg, and the Department of Psychiatry, University of Heidelberg. Data from 33 patients had been analyzed in a previous study (34). Psychopathology was rated by means of the Scale for the Assessment of Positive Symptoms (SAPS) (44), the Scale for the Assessment of Negative Symptoms (SANS) (45), the Brief Psychiatric Rating Scale (BPRS) (46), and the Apathy Evaluation Scale (AES) (47), respectively.

Twenty-one healthy comparison subjects (53.67 ± 8.00 years of age) were recruited through newspaper advertisement. All groups were closely matched (*p* > 0.20) with regard to sex (% male: older patients: 76.00, younger patients 60.90, healthy controls 57.10) and education (years of education: older patients 13.28 ± 3.30, younger patients 12.61 ± 1.80, healthy controls 13.90 ± 2.12); in addition, healthy control subjects and older patients were matched for age (*p* > 0.80). All subjects were right-handed (48). Exclusion criteria for all participants were: (1) a lifetime history of neurological disorder, head injury, or substance dependency, (2) an axis II disorder, (3) mental retardation, (4) being not fluent in German. The study was approved by the ethics committee of the medical faculty of Heidelberg University and all participants gave written informed consent after the procedures of the study had been fully explained.

### Neurocognitive assessment

Autobiographical memory performance was assessed using a semi-structured autobiographical interview (49), adapted from Kopelman et al. (50). Autobiographical events and facts from the following five lifetime periods were addressed: preschool (up to 6 years of age), primary school (from 6 to 11 years of age), secondary school (from 11 years of age to graduation), early adulthood (from graduation to 35 years of age), and recent 5 years. From each lifetime period, the participants were asked to report details of one autobiographical event (max. 11 points) and five autobiographical facts (max. 5 points). The scoring parameters and the control for delusional memories are described elsewhere in detail (34). In consideration of the younger patient group, we restricted our analyses to memories from four lifetime periods. Our analyses refer to the total score of the remembered details (max. 44 points) and the total score of personal facts (max. 20 points); in case of remote memories, we added scores from lifetime periods 1 and 2 (preschool and primary school); for recent memories, we added scores from lifetime periods 3 and 4 (secondary school and early adulthood); respectively. The interview shows a sufficient internal consistency (Cronbach’s α) of the two scales. In a previous study, the inter-rater reliability ranged from 0.954 to 0.979 (51).

A comprehensive neuropsychological test battery including tests of verbal memory, short-term and working memory, information processing speed, executive functioning as well as remote semantic memory was administered to each subject. Verbal memory was tested with the subtests Logical Memory I (immediate recall) and II (delayed recall) from the Wechsler memory scale-revised (WMS-R) (52); short-term and working memory were evaluated using the Digit Span forward and backward from the WMS-R. The assessment of processing speed and executive function was based on Trail Making Test (TMT) version A and B (53). Remote semantic memory was tested via recognition performance of famous people using the Bielefelder Famous Faces Test (BFFT) (54).

The completion of the whole test battery took approximately 3 h and was organized in two or more sessions.

### MRI data acquisition

The magnetic resonance imaging (MRI) data were obtained at the German Cancer Research Centre using a 3.0 Tesla scanner (SIEMENS MAGNETOM TrioTim syngo MR B15). A high-resolution T1-weighted magnetization prepared rapid gradient echo (MP-RAGE) sequence was performed with the following acquisition parameters: 160 sagittal slices, voxel size = 1.0 mm × 1.0 mm × 1.0 mm, image matrix = 256 × 256, flip angle 9°, TR = 2300 ms, TE = 2.98 ms, TI = 900 ms.

### MRI data analysis

Magnetic Resonance Imaging data analysis was carried out with FSL (FMRIB Software Library v 4.1.7, Oxford Centre for Functional MRI of the Brain) (55, 56). FMRIB’s Integrated Registration and Segmentation Tool (FIRST v 1.2) was used to segment and analyze volumes of left and right hippocampus (57). The segmentation routine FIRST is a mesh model based tool for segmenting subcortical brain structures utilizing a library of manually segmented images obtained from the Center for Morphometric Analysis, Massachusetts General Hospital, Boston. Additional information about this method is given elsewhere (34, 58). The hippocampus volume comprised the dentate gyrus, the ammonic subfields (CA1-4), the prosubiculum, and the subiculum [cf. (58, 59)]. After segmentation of the hippocampus, which is based on shape models and voxel intensities, the structure was divided in an anterior and a posterior section by the coordinates of the center of gravity (posterior: Y axis >0, anterior: Y axis <0) (59). Results were given in mm^3^; for volumetric calculations, default parameters were used.

The calculation of intracranial volume (ICV) was conducted by summing-up the volumes of gray matter, white matter, and cerebrospinal fluid arrived by using the T1-weighted scans via SPM5 software (http://www.fil.ion.ucl.ac.uk/spm).

### Statistical analysis

SPSS version 17.0. was used for statistical analysis; in all comparisons, the alpha level was set at *p* < 0.05. Group differences in demographic and clinical characteristics were investigated via univariate analysis of variance (ANOVA), independent group *t*-test, or χ^2^-test, respectively. Repeated measures ANOVAs with group (younger vs. older patients vs. healthy subjects) as between-subject factor and period as within-subject factor (all lifetime periods: 1–4, early periods: 1–2, late periods: 3–4) were conducted for the AM scores. Greenhouse-Geisser correction (60) was applied in case of violation of sphericity. The additional neuropsychological data were subjected to multivariate analysis of variance (MANOVA) with group as between-subject factor. Volumetric group differences were also calculated via MANOVA with ICV as covariate, which accounts for potential differences in premorbid brain size and hence considers brain size associated variance. *Post hoc* analyses were performed by using the Bonferroni formula; in case of violation of the homogeneity of variance assumption, the Games–Howell test was applied [(61) p. 134]. Partial correlations (two-tailed) were examined to analyze associations between hippocampal volume and AM performance and neuropsychological scores, respectively. These analyses were done for healthy controls and patients separately, the two patient groups were merged. We adjusted for the effects of educational level due to possible influences of this variable on cognitive performance.

## Results

### Clinical characteristics

As described above, the two patient groups differed significantly with respect to age and as a matter of fact with respect to duration of illness (*p* ≤ 0.000) –as age at illness onset differed not significantly between the groups (*p* > 0.20) – with a mean duration of illness (calculated since initial diagnosis) of 31 years and 11 years, respectively (Table 1). The majority of the older patients (68%) live in psychiatric long-term units in contrast to about 35% of the younger patients (χ^2^ = 5.30; *p* = 0.02; df = 1). Regarding antipsychotic medication indicated in chlorpromazine (CPZ) equivalents (62), the younger patients received significantly higher doses than the older patient group (*p* < 0.05). Symptom severity differences were found with respect to BPRS score, which is significantly higher in younger than in older patients (*p* < 0.03). Negative symptoms predominated in both patient groups (*p* ≤ 0.02).

**Table 1:** **Clinical characteristics of the patients**.

	Older patients	Younger patients	*T* (df)^a^	*p*
	*N* = 25	*N* = 23	
	*M* (SD)	*M* (SD)	
Duration of illness, years	31.36 (10.54)	10.48 (6.40)	8.22 (46)	**0.000**
Age at illness onset, years	24.56 (8.63)	22.30 (4.73)	−1.13 (37, 84)	0.264
Hospitalized (%)	68.00	34.78		**0.021^b^**
CPZ equivalents, mg	525.06 (395.38)	859.26 (681.76)	−2.05 (34, 68)	**0.048**
Antipsychotic medication, AT/AT + T/T/no medication, *N*	11/9/2/3	17/5/1/0		0.128^b^
Additional antidepressive medication, *N*	10	10		0.807^b^
Additional benzodiazepines, *N*	2	1		0.576^b^
BPRS	35.28 (9.21)	41.43 (9.07)	2.33 (46)	**0.024**
SAPS	13.92 (14.69)	19.48 (16.42)	−1.24 (46)	0.222
SANS	31.08 (20.65)	34.83 (25.60)	−0.56 (46)	0.578
AES	26.52 (10.40)	23.04 (11.83)	1.08 (46)	0.284

*AES, Apathy Evaluation Scale; AT, atypical antipsychotics; AT + T, atypical and typical antipsychotics; BPRS, Brief Psychiatric Rating Scale; CPZ, chlorpromazine; SANS, Scale for the Assessment of Negative Symptoms; SAPS, Scale for the Assessment of Positive Symptoms; T, typical antipsychotics*.

*^a^*t*-test (df, degrees of freedom)*.

*^b^χ^2^-test*.

### Hippocampal volume

As hypothesized, the MANOVA revealed significant group differences with respect to left and right hippocampus, as well as the respective subregions of anterior and posterior sections (Table 2). *Post hoc* tests showed reduced hippocampal volumes of the older patient group in comparison to the age-matched healthy control group (0.000 ≤ *p* < 0.04) and to the younger patients (0.000 ≤ *p* < 0.02). In case of right anterior hippocampus, this difference was significant for older in contrast to younger patients (*p* = 0.007), with a trend-level significant difference to healthy subjects (*p* = 0.066).

**Table 2:** **Hippocampal volumes (mm^3^) of patients and healthy controls**.

	Older patients	Younger patients	Healthy controls	*F* (df)^a^	*p*	Partial η^2^	Post hoc^b^
	*N* = 25	*N* = 23	*N* = 21	
	*M* (SD)	*M* (SD)	*M* (SD)	
Left hippocampus	3377.24 (593.49)	3997.91 (358.62)	3924.95 (381.19)	16.22 (2, 65)	**0.000**	0.333	**O < Y, H**
Right hippocampus	3659.24 (688.05)	4079.44 (397.97)	4001.19 (392.20)	5.52 (2, 65)	**0.006**	0.145	**O < Y, H**
Left anterior hippocampus	1421.84 (244.16)	1674.52 (158.60)	1647.48 (197.75)	13.14 (2, 65)	**0.000**	0.288	**O < Y, H**
Left posterior hippocampus	1955.40 (357.44)	2323.39 (219.75)	2277.48 (198.87)	16.91 (2, 65)	**0.000**	0.342	**O < Y, H**
Right anterior hippocampus	1539.08 (273.44)	1717.83 (144.25)	1665.24 (187.77)	5.44 (2, 65)	**0.007**	0.143	**O < Y**
Right posterior hippocampus	2120.16 (420.42)	2361.61 (266.41)	2335.95 (221.95)	5.24 (2, 65)	**0.008**	0.139	**O < Y, H**

*H, healthy controls; O, older patients, Y, younger patients*.

*^a^MANOVA, ICV as covariate (df, degrees of freedom)*.

*^b^Bonferroni (5%)*.

The volume decrease of the older patients – based on 100% in the control group – accounts for 13.95% in left and 8.55% in right hippocampus.

No significant correlations of hippocampal volumes with CPZ equivalents or psychopathological symptom scores arose (*p* > 0.09). When controlled for age, the correlations between left and right hippocampal volumes and duration of illness were not significant (*p* > 0.50). Repeated analyses based on relative hippocampal volumes (quotient of hippocampal volume and ICV) yielded concurrent results.

### Autobiographical memory and neuropsychological performance

**Figure 1:**
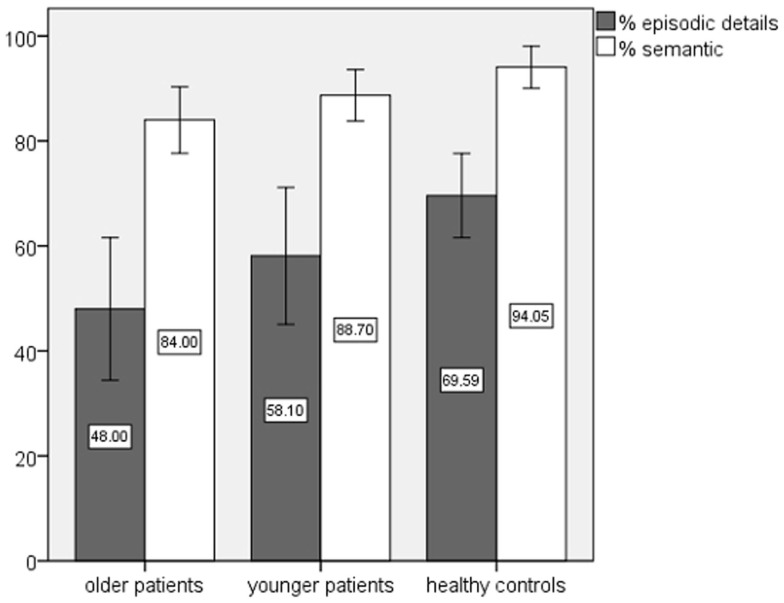
**Autobiographical memory performance of patients and healthy controls in percent**.

The repeated measures ANOVAs yielded a significant main effect of group for episodic details and semantic AM from four lifetime periods (Table 3). Younger and older patients achieved comparable performances in both AM scores (*p* > 0.40). However, *post hoc* tests showed significant impairments of the older patient group in comparison to the healthy subjects concerning episodic (*p* = 0.018) and semantic AM (*p* = 0.022). In Figure 1, the AM performance of patients and healthy controls is given in percent – based on 100% for the maximal score.

**Table 3:** **Autobiographical memory performance of patients and healthy controls**.

	Older patients	Younger patients	Healthy controls	*F* (df)^a^	*p*	Partial η^2^	Post hoc^b^
	*N* = 25	*N* = 23	*N* = 21	
	*M* (SD)	*M* (SD)	*M* (SD)	
Episodic details, periods 1–4	21.12 (14.47)	25.57 (13.26)	30.62 (7.76)	3.41 (2, 66)	**0.039**	0.094	**O < H**
Episodic details, periods 1–2	10.28 (7.66)	12.43 (7.22)	12.90 (5.34)	1.01 (2, 66)	0.371	0.030	
Episodic details, periods 3–4	10.84 (8.11)	13.13 (7.25)	17.71 (4.46)	5.80 (2, 66)	**0.005**	0.149	**O, Y < H**
Semantic, periods 1–4	16.80 (3.07)	17.74 (2.26)	18.81 (1.75)	3.81 (2, 66)	**0.027**	0.103	**O < H**
Semantic, periods 1–2	7.64 (2.10)	8.13 (1.79)	9.00 (1.30)	3.35 (2, 66)	**0.041**	0.092	**O < H**
Semantic, periods 3–4	9.16 (1.34)	9.61 (0.78)	9.81 (0.60)	2.66 (2, 66)	0.077	0.075	

*H, healthy controls; O, older patients, Y, younger patients*.

*^a^Repeated measures ANOVA (df, degrees of freedom)*.

*^b^Games–Howell Test (5%)*.

The effect of period reached significance for episodic (*p* < 0.02; *F* = 4.05; partial η^2^ = 0.06; df = 2.69) and semantic AM (*p* ≤ 0.000; *F* = 29.91; partial η^2^ = 0.31; df = 2.59). *Post hoc* tests revealed better performances for recent in contrast to remote memories (period 1 vs. period 4 *p* ≤ 0.001; period 2 vs. period 4 *p* ≤ 0.05).

The interaction group × period indicated in case of semantic AM a non-significant effect (*p* > 0.25), in case of episodic AM a trend level significance (*p* = 0.07; *F* = 2.01; partial η^2^ = 0.06; df = 5.38), with raw scores showing a recency effect in the control group. In both patient groups, the recency effect was less pronounced and the performance for the periods 2 and 3 in the older patient group (period 2 in the younger patients) was reduced in comparison to period 1.

Separate analyses for recent and remote lifetime periods (period 1 and 2 vs. period 3 and 4) yielded no significant main or interaction effects for remote episodic AM (*p* > 0.20), in contrast to recent episodic AM, which showed a significant effect for group. Healthy control subjects outperformed older (*p* = 0.002) and younger patients (*p* < 0.04), with no significant difference between the patient groups (*p* > 0.50). The analysis of remote semantic memories revealed a significant effect of group, showing that the older patients reported fewer autobiographical facts than their healthy counterparts (*p* < 0.03), again the difference between the patient groups did not reach significance (*p* > 0.60). In case of recent semantic memories, no significant effects were found (*p* ≥ 0.07).

Episodic but not semantic AM was significantly correlated with education in the patient group (*r* = 0.30; *p* < 0.04; df = 48). Apart from this, episodic and semantic AM sumscores were not significantly correlated with age, duration of illness, and CPZ equivalents (*p* > 0.10), but episodic AM was significantly negatively correlated with SANS (*r* = −0.41; *p* = 0.004; df = 48) and AES sumscores (*r* = −0.33; *p* = 0.02; df = 48).

**Figure 2:**
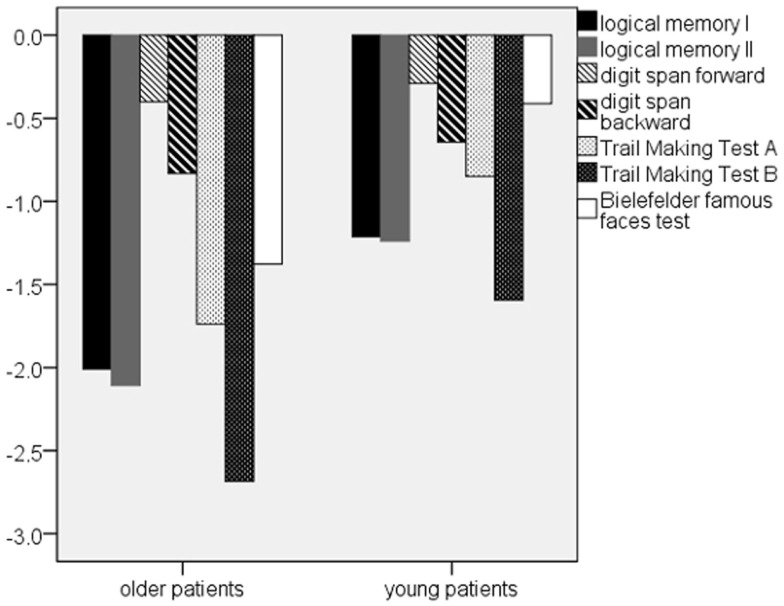
**Patients’ *z*-scores of the additional neuropsychological parameters**. Note: *z*-scores are based on WMS-R/TMT, in case of BFFT on healthy control data.

With regard to the additional neuropsychological parameters, the older patient group performed significantly worse in tests measuring verbal memory (Logical Memory I and II), information processing speed, and executive functions (TMT A and B), and remote semantic memory (BFFT) as well (0.000 ≤ *p* ≤ 0.007). No significant group differences were found in short-term and working memory (Table 4; Figure 2). In case of Logical Memory (*p* ≤ 0.000) and TMT B (*p* = 0.05) also, the younger patients showed marked impairments in comparison to the even older healthy control group, with performance levels between the older patients and the healthy probands.

**Table 4:** **Neuropsychological performance of patients and healthy controls**.

	Older patients	Younger patients	Healthy controls	*F* (df)^a^	*p*	Partial η^2^	Post hoc^b^
	*N* = 25	*N* = 23	*N* = 21	
	*M* (SD)	*M* (SD)	*M* (SD)	
Logical memory I	14.60 (6.60)	21.04 (8.85)	29.95 (5.12)	27.02 (2, 66)	**0.000**	0.450	**O < Y < H**
Logical memory II	9.96 (5.86)	16.30 (7.90)	25.71 (5.03)	34.73 (2, 66)	**0.000**	0.513	**O < Y < H**
Digit span forward	6.88 (1.92)	7.43 (1.73)	7.81 (2.14)	1.36 (2, 66)	0.264	0.040	
Digit span backward	5.16 (1.80)	5.74 (1.79)	6.38 (1.69)	2.74 (2, 66)	0.072	0.077	
Trail making test A	61.63 (41.66)	36.52 (15.85)	34.76 (12.11)	7.09 (2, 66)	**0.002**	0.177	**O > H, Y**
Trail making test B	188.25 (62.63)	101.17 (59.41)	68.48 (21.56)	32.77 (2, 66)	**0.000**	0.498	**O > Y > H**
Bielefelder famous faces test^c^	2.10 (0.38)	2.33 (0.48)	2.43 (0.24)	3.92 (2, 57)	**0.025**	0.121	**O < H**

*H, healthy controls; O, older patients, Y, younger patients*.

*^a^MANOVA (df, degrees of freedom)*.

*^b^Bonferroni (5%), TMT B and BFFT: Games–Howell Test (5%)*.

*^c^Reduced *N* in groups: 23 older patients, 20 younger patients, 17 healthy controls*.

No significant correlations between neuropsychological parameters and CPZ-equivalents emerged (*p* > 0.05). Significant correlations arose between level of education and “Logical Memory I,” “Digit Span backward” (0.38 < *r* < 0.44; 0.001 < *p* < 0.006; df = 48), “TMT B” (*r* = −0.41; *p* = 0.004; df = 48), and “BFFT” (*r* = 0.41; *p* = 0.006; df = 43). “Logical Memory I and II” (−0.49 < *r* < −0.58; *p* ≤ 0.000; df = 48), “TMT A and B” (0.30 < *r* < 0.36; *p* ≤ 0.03; df = 48), and “BFFT” (*r* = −0.32; *p* = 0.04; df = 43) were significantly correlated with SANS sumscore. Accordingly, we found significant correlations between AES sumscore and “Logical Memory I and II” (−0.46 < *r* < −0.56; *p* ≤ 0.001; df = 48), “Digit Span backward” (*r* = −0.36; *p* = 0.01; df = 48), and “TMT A and TMT B” (0.28 < *r* < 0.40; 0.005 <*p* < 0.05; df = 48). SAPS and BPRS sumscores were not significantly correlated with the neuropsychological parameters (*p* > 0.10), with the exception of BFFT, which showed a significant correlation with SAPS sumscore (*r* = 0.33; *p* = 0.03; df = 43).

### Correlations between hippocampal volume and autobiographical memory

In the patient group as a whole, correlational analyses between hippocampal volumes and AM revealed several significant relationships, as can be seen in Table 5. Episodic AM performance was significantly correlated with left (*r* = 0.29; *p* = 0.05) and especially left anterior (*r* = 0.31; *p* < 0.04) hippocampal volume. Further analyses showed that this association can mainly be explained by recent autobiographical episodic details (lifetime period 3–4), which were significantly correlated with left anterior hippocampus (*r* = 0.30; *p* < 0.05). On the other hand, semantic autobiographical memories from the four lifetime periods were significantly associated with left anterior and posterior hippocampal volumes independent of their recency (0.29 < *r* < 0.31; 0.03 < *p* < 0.05).

**Table 5:** **Partial correlations^a^ between hippocampal volume and autobiographical memory in the patient group**.

Hippocampus		Left	Right	Left anterior	Left posterior	Right anterior	Right posterior
**Autobiographical memory**
Episodic details, periods 1–4	r^b^	**0.285**	0.223	**0.308**	0.261	0.242	0.205
	*p*	**0.052**	0.133	**0.035**	0.076	0.101	0.166
Episodic details, periods 1–2	*r*	0.251	0.161	0.273	0.229	0.201	0.133
	*p*	0.089	0.279	0.064	0.121	0.177	0.374
Episodic details, periods 3–4	*r*	0.269	0.244	**0.289**	0.247	0.241	0.241
	*p*	0.068	0.098	**0.049**	0.093	0.102	0.103
Semantic, periods 1–4	*r*	**0.296**	0.252	**0.307**	0.281	0.252	0.246
	*p*	**0.043**	0.088	**0.036**	0.056	0.087	0.096
Semantic, periods 1–2	*r*	0.250	0.218	**0.293**	0.214	0.228	0.206
	*p*	0.090	0.142	**0.046**	0.149	0.123	0.164
Semantic, periods 3–4	*r*	0.280	0.229	0.231	**0.305**	0.212	0.235
	*p*	0.057	0.122	0.119	**0.037**	0.152	0.113

*^a^Controlled for years of education*.

*^b^Pearson product-moment correlation coefficient (two-tailed) *p* ≤ 0.05, df = 45*.

In the healthy control group, left anterior and posterior hippocampus were significantly associated with semantic autobiographical memories, especially with memories from childhood periods (0.40 < *r* < 0.60; 0.005 < *p* < 0.05). In contrast, the episodic autobiographical memories showed no significant correlations with hippocampal volume (*p* ≥ 0.09) (Table 6).

**Table 6:** **Partial correlations^a^ between hippocampal volume and autobiographical memory in the healthy control group**.

Hippocampus		Left	Right	Left anterior	Left posterior	Right anterior	Right posterior
**Autobiographical memory**
Episodic details, periods 1–4	r^b^	0.289	0.331	0.305	0.250	0.315	0.314
	*p*	0.217	0.154	0.191	0.288	0.176	0.177
Episodic details, periods 1–2	*r*	0.183	0.174	0.210	0.141	0.214	0.124
	*p*	0.440	0.464	0.374	0.552	0.366	0.602
Episodic details, periods 3–4	*r*	0.279	0.363	0.275	0.262	0.288	0.394
	*p*	0.233	0.115	0.241	0.264	0.218	0.085
Semantic, periods 1–4	*r*	**0.491**	0.393	**0.455**	**0.488**	0.394	0.357
	*p*	**0.028**	0.087	**0.044**	**0.029**	0.086	0.123
Semantic, periods 1–2	*r*	**0.567**	0.426	**0.529**	**0.560**	0.430	0.384
	*p*	**0.009**	0.061	**0.017**	**0.010**	0.058	0.095
Semantic, periods 3–4	*r*	0.222	0.235	0.200	0.227	0.229	0.218
	*p*	0.346	0.319	0.398	0.336	0.331	0.355

*^a^Controlled for years of education*.

*^b^Pearson product-moment correlation coefficient (two-tailed) *p* ≤ 0.05, df = 18*.

### Correlations between hippocampal volume and neuropsychology

Several significant correlations between particularly right hippocampal volume and the additional neuropsychological parameters of verbal memory, working memory, and executive functions emerged (Table 7). Verbal memory scores were found to significantly correlate with right anterior and posterior hippocampal volumes (0.25 < *r* < 0.35; 0.01 < *p* < 0.05). In addition, left and right anterior hippocampus showed significant positive associations with working memory (0.25 < *r* < 0.35; 0.02 < *p* < 0.05). TMT B had broad significant negative relationships with left and right anterior and posterior hippocampus (−0.40 < *r* < −0.50; 0.000 < *p* < 0.005). No significant associations with hippocampal volume could be reported for short-term memory, processing speed (TMT A), and remote semantic memory (BFFT) (*p* > 0.10).

**Table 7:** **Partial correlations^a^ between hippocampal volume and neuropsychological parameters in the patient group**.

Hippocampus		Left	Right	Left anterior	Left posterior	Right anterior	Right posterior
**Neuropsychology**
Logical memory I	r^b^	0.201	**0.330**	0.223	0.180	**0.344**	**0.314**
	*p*	0.175	**0.023**	0.131	0.225	**0.018**	**0.031**
Logical memory II	*r*	0.155	**0.294**	0.194	0.124	**0.321**	0.271
	*p*	0.297	**0.045**	0.192	0.405	**0.028**	0.066
Digit span forward	*r*	0.201	0.087	0.204	0.193	0.113	0.068
	*p*	0.176	0.563	0.169	0.194	0.451	0.649
Digit span backward	*r*	0.260	**0.291**	**0.319**	0.213	**0.325**	0.264
	*p*	0.077	**0.047**	**0.029**	0.151	**0.026**	0.073
Trail making test A	*r*	−0.181	−0.141	−0.193	−0.167	−0.142	−0.137
	*p*	0.224	0.345	0.193	0.262	0.341	0.359
Trail making test B	*r*	−**0.446**	−**0.426**	−**0.466**	−**0.419**	−**0.412**	−**0.427**
	*p*	**0.002**	**0.003**	**0.001**	**0.003**	**0.004**	**0.003**
Bielefelder famous faces test^c^	r^d^	0.073	0.030	0.098	0.054	0.054	0.014
	*p*	0.647	0.851	0.538	0.736	0.734	0.930

*^a^Controlled for years of education*.

*^b^Pearson product-moment correlation coefficient (two-tailed) *p* ≤ 0.05, df = 45*.

*^c^Reduced *N* in groups: 23 older patients, 20 younger patients*.

*^d^Product-moment correlation coefficient (two-tailed) *p* ≤ 0.05, df = 40*.

In the healthy control group, the correlations between hippocampal volume and neuropsychological parameters failed significance (*p* > 0.05).

## Discussion

The present study yielded the following main findings, which are discussed in the subsequent sections: (1) a pronounced hippocampal volume reduction in older patients with schizophrenia, which is also detectable albeit to a lesser extent in younger patients with a chronic course of the disease; (2) AM performance for recent episodic details is significantly reduced in older and younger patients in contrast to healthy control subjects; neuropsychological deficits of the patients, i.e., verbal memory and executive functions, seem to deteriorate in the course of the disease; (3) hippocampal volume is significantly correlated with episodic and semantic AM, with different correlation patterns in patients and healthy controls; (4) only in the patient group, significant correlations between hippocampal volume and verbal memory, working memory, and executive functions arose.

### Clinical characteristics

Most of the older patients were – in contrast to the younger patients – chronically hospitalized and therefore in a more remitted phase of the disorder. The significantly higher doses of CPZ equivalents in the younger patient group can therefore be explained by higher psychiatric symptom severity (BPRS sumscore) in this group, due to a shorter interval since last acute phase. Moreover, reduced CPZ equivalents in the older patient group can additionally be explained by age-related pharmacokinetic and pharmacodynamic changes (63–65). Considering the chronic course of the disease in our patient samples, predominant negative symptoms in both patient groups were expected.

### Hippocampal volume

As expected, significant hippocampal volume alterations were evident between the groups, i. e., left and right hippocampal volumes were reduced in the older patients in comparison to the healthy subjects. These volumetric reductions of 9–14% are in line with previous studies, which focused on older patients with a duration of the disease of 25–30 years (22, 26, 27). Corresponding to the results of Weiss et al. (22), we did not find a selective volumetric reduction along the anterior–posterior hippocampal axis. Similarly, the older patients’ left and right hippocampal volumes were significantly reduced in comparison to the younger patient group. These results may reflect normal age-related brain changes on the one hand (66–68) and disease-related processes on the other hand (69, 70). For example, in a review of 11 longitudinal studies, the authors reported progressive cerebral changes (especially in frontal and temporal areas) in patients with chronic schizophrenia with a volume reduction of −0.5%/year, which was more than twice the tissue decrease in healthy subjects (−0.2%/year) (71).

Hippocampal atrophy early in the disease process was previously described (72, 73), and is indirectly supported by the present findings, which show similar hippocampal volumes in younger patients and healthy subjects, which were 20 years older on average. Likewise, Chakos et al. (74) reported a significant reduction of hippocampal volume in younger (24.9 ± 8.8 years of age) and older patients (37.1 ± 11.8 years of age) in comparison to healthy controls, an effect which was more pronounced in the older patient group.

In our patient group, no significant interactions arose between hippocampal volume and CPZ-equivalents, sumscores of psychopathology, or duration of illness. Nevertheless, potential effects of especially typical antipsychotic medication on brain structure have to be considered, particularly in samples of patients with chronic schizophrenia as in our study, and may primarily affect basal ganglia volume (75–77). Although not all patients in the present study were exclusively treated with atypical antipsychotics, it should be noted that no extrapyramidal side-effects such as tardive dyskinesia could be observed. Recently, Ho et al. (78) reported in a longitudinal study with 211 first-episode patients a significant effect of antipsychotic medication on brain volume while controlling for severity of psychopathology, duration of follow-up, and substance abuse. Gray matter volume reduction in multiple cerebral areas was associated with higher doses independent of type of medication. However, no significant effects of antipsychotic type (22, 28, 79 80), duration of treatment (10), or dosage of medication (10, 81–84) on hippocampal volume were detected in patients with first-episode or chronic schizophrenia. Moreover, treatment with atypical antipsychotics was associated with larger hippocampal volumes than treatment with typical antipsychotics (74, 85). Longitudinally, the cumulative intake of atypical antipsychotic medication was related to a smaller hippocampal decrease in a sample of patients with chronic schizophrenia. This effect was – trend level only – reversed for typical medication (86).

In agreement with the present results (26) found, no significant correlations between hippocampal volume and duration of illness or severity of psychopathology (BRPS, SAPS, SANS) in a sample of middle-aged patients with chronic schizophrenia [see also Ref. (87)]. Other studies reported consistently with our findings no significant associations between hippocampal volume and illness duration (11, 82, 88, 89). Positive correlations between severity of psychopathology and right hippocampal volume were previously described (82), though there are also negative findings (28, 83). While positive symptoms seem to be related to hippocampus (90–92), in our patient samples negative symptoms were predominating.

### Autobiographical memory and neuropsychological performance

As hypothesized, AM performance was reduced in the older patients in comparison to the healthy control group. These differences applied for episodic and semantic autobiographical memories as well. Regarding different lifetime periods, significant impairments of the older patients were found with respect to recent episodic and remote semantic memories. In case of recent episodic memories also, the younger patient group was impaired in contrast to the even older healthy control group. A main effect of period indicates better memory performance for recent than remote autobiographical memories. The interaction group × period shows a tendency for episodic AM impairment in the patients especially for periods after onset of the disease.

The assessment of AM without temporal limitation and the differentiated profiles of episodic and semantic memories rule out confounding effects like reduced cognitive capacities, e. g., information processing speed or reduced motivation in our patient groups [cf. (31)]. The potential impact of confabulations (93) is considerably weakened by the fact that mnestic deficits were clearly evident in the autobiographical interview. Previous studies on AM in patients with schizophrenia found no evidence of false memories or confabulations (32, 94). Studies investigating susceptibility to false memories in patients with schizophrenia did not report elevated false recognition errors or intrusions (95–98). Concerning the truthfulness of the reported autobiographical memories, an additional interviewing of relatives is difficult not only in terms of practicability but also reduces the eligible episodic memories to shared experiences. Moreover, episodic autobiographical memories reflect subjective impressions, so the personal meaning of an event can nonetheless be accurate (30).

Our results are consistent with previous studies showing diminished episodic and semantic AM in younger patients with chronic schizophrenia, with reduced specificity, and detail of the reported autobiographical episodes, recency effect, and memory deficits especially from the time of illness onset (29, 30, 31, 33, 94). In supplementing and extending our previous results (99), the younger patients remembered fewer recent episodic details than the healthy control group, while there was no significant difference between the patient groups. When interpreting these results, it has to be considered that the younger patients have a shorter time interval to autobiographical events from different lifetime periods due to their lower age, which emphasizes the reported deficits.

No significant correlations arose between sumscores of episodic and semantic AM and duration of illness or CPZ-equivalents, which corresponds to previous studies (30, 94). In accordance with findings from Corcoran and Frith (100) and Harrison and Fowler (101), the episodic sumscore was inversely correlated with SANS and AES scores; however, relationships with psychiatric symptoms were not consistently reported (94).

Negative symptoms are associated with frontal-executive processes (102, 103), which can be one possible explanation for the AM impairment given a sample of patients with predominantly negative symptoms (cf. impaired executive functions in our patient groups, discussed below). Similarly, executive functions like coordination and organization of information are among the best predictors for deficits of long-term memory in patients with schizophrenia (104). Likewise, in patients with depression, a positive association between specificity of AM and central executive processes of working memory was described (105); similar findings apply to healthy older adults (106).

Autobiographical specificity has also been discussed in light of context memory (105, 107), which is impaired in patients with schizophrenia (108). The reduced ability to combine contextual cues of events together (e. g., source, time) to form a coherent memory representation results in a more fractionated recollection of those events and in reduced autonoetic consciousness (109). Similarly, meta-analytic findings indicate a significant more pronounced deficit in associative recognition (intact vs. rearranged pairings of item with item/source/temporal order – therefore depending on conscious recollection) relative to item recognition (old/new judgment) in patients with schizophrenia (110). Therefore, AM deficits in schizophrenia are not only characterized by reduced specificity but also by reduced conscious recollection (32). In this respect, the critical role of the hippocampus for recollection rather than familiarity was emphasized by Yonelinas (111) and Eichenbaum et al. (112).

As expected, the older patients showed a significantly reduced performance in comparison to the healthy control subjects in all additional neuropsychological parameters with exception of short-term and working memory.

Dysfunction of verbal declarative memory is a very robust empirical finding in schizophrenia (3, 4, 113) and one of the strongest predictors of functional outcome (114). Other cognitive parameters as attention deficits or symptom fluctuations cannot fully explain the extent of verbal declarative memory deficits in schizophrenia (3, 115).

Pronounced impairments in executive functions are among the most consistently reported cognitive deficits in schizophrenia and affect – together with reduced processing speed – memory performance (4, 113, 116–118).

The BFFT was applied to examine remote semantic memory, i. e., context-free knowledge without personal relevance in contrast to autobiographical episodic and semantic memory. Corresponding to our results, deficits in identifying and naming famous faces were documented in patients with chronic schizophrenia (119, 120).

In contrast to deficits of recent episodic AM, which were not significantly different between the two patient groups, the younger patients outperformed the older patients as expected in a range of neuropsychological parameters including verbal memory, processing speed, and executive functions. In case of verbal memory and executive functions, the largest group differences were evident, with the younger patients showing marked deficits even in comparison to the older healthy controls. The differentiating profiles of AM and additional neuropsychological domains between the three groups examined emphasize once again the exceptional position of AM as probably the most complex memory system (121).

Logical memory and TMT B performance of the older patient group in our study are comparable with that of patients with mild cognitive impairment (122, 123). Given hippocampal changes in patients with schizophrenia – which even can show a similar amount of volume reduction as in patients with Alzheimer’s disease (28) – these results raise the question of neurodegenerative processes in schizophrenia (124). However, cognitive profiles of patients with schizophrenia and Alzheimer’s disease are different in spite of pronounced impairments in both groups (125–128). Additionally, a meta-analysis (129) and several subsequent studies did not report an Alzheimer’s disease-like neuropathology in schizophrenia (130–135). However, the lacking evidence of typical neurodegeneration in schizophrenia does not exclude progressive components like changes in neuropsychological functioning or brain volume (124). Furthermore, as in our sample, age-related cognitive deterioration can be suggested in older, institutionalized individuals with chronic schizophrenia (136). In this context, it has to be mentioned that physical illnesses like the metabolic syndrome, which are associated with cognitive deficits and increase with rising age (137), are more prevalent among patients with schizophrenia (138, 139).

A similar pattern of cognitive deterioration with increasing chronicity of the disease as in our sample was observed in a study with 122 patients with chronic schizophrenia (140). As in our results, among others, especially verbal memory was affected by duration of illness, therefore, suggesting a progressive deteriorating course, while other neurocognitive domains remain rather stable. These results are well in line with findings of a recent meta-analysis, which reports cross-sectionally large and heterogeneous deficits in global cognition (*d* = −1.19) and specific neuropsychological domains as language, memory, and executive functions (*d* = −0.7 to −1.14) in older individuals with chronic schizophrenia (141). According to the results presented here, advanced age and living institutionalized were associated with more severe impairments; antipsychotic dose was not a significant moderator.

Consistent with results of meta-analyses and reviews, only non-significant correlations between additional neuropsychological parameters and CPZ-equivalents arose (3, 115, 116). Other studies reported evidence of beneficial impact of atypical (142–144) and typical (145–147) antipsychotics on cognitive abilities in patients with schizophrenia.

In our study, negative symptoms (SANS sumscore) were significantly correlated with diminished performance of Logical Memory, TMT A and B, and BFFT. This also applies to the extent of apathy, while similar associations of positive symptoms or BPRS sumscore with neuropsychological performance did not appear. In accordance with our results, associations between negative symptoms and memory or executive functions were repeatedly reported (3, 115, 116, 148).

### Correlations between hippocampal volume and autobiographical memory

Significant correlations between AM and hippocampal volume arose in the patient and control group as expected. Episodic and semantic AM was associated with left hippocampus in the patient group. Especially, recent episodic memories were correlated with hippocampus; however, in case of semantic memories these correlations were significant for both recent and remote memories. In contrast, in the control group the correlations were restricted to remote semantic AM. This pattern can only partly be explained by the standard model of consolidation, which postulates a time-limited role of hippocampus in the formation and retention of declarative memory (36, 37, 149). This is true for episodic AM in the patient group; however, in case of semantic AM there was no temporal gradient; hence also the multiple-trace-theory cannot account for these results (38, 40). According to this view, maintenance and re-experiencing of vivid and detailed events depend on hippocampal formation as long as they exist, whereas semantic, that is context-free memories and schematic representations, benefit from hippocampal contribution before they are retrieved independent of this structure.

In case of the present study, the frequency of retrieval of the semantic information addressed in our interview and especially the frequency of retrieval of recent semantic knowledge may be reduced in patients with schizophrenia due to diminished necessity. This is of particular importance as more seldomly retrieved memories remain dependent on hippocampus no matter how old they are (150, 151). This assumption may also apply to the correlation between remote semantic AM and hippocampus in the control group. In healthy subjects, questions relating to former addresses or names of teachers possibly refer to facts more seldomly retrieved than episodic memories like the own wedding. It might be argued that patients with chronic schizophrenia experienced, due to their illness, fewer “normative” autobiographical events like wedding which could be reported. However, apart from that, all episodes were scored according to the remembered details. In contrast, one might expect that healthy persons have frequently reported such common events, which therefore are semantizised, and exist independent of hippocampal formation, as our results indicate (152).

To test the influence of retrieval frequency of remote semantic memory, we used the BFFT. Participants had to identify famous faces known from the media. Results show only non-significant associations between BFFT-performance and hippocampal volume in both groups. Additional indications for the assumption that frequently retrieved remote semantic memories are rather independent of hippocampus came from previous studies (153–155). In this context, the personal relevance of semantic information has to be considered as a potential factor that may result in a performance advantage (156, 157). However, the autobiographical significance of the stimuli used in the BFFT was not explicitly requested as the recognition was focused on.

Another objection also concerns the retrieval frequency of remote semantic AM, which as a matter of fact should have been frequently retrieved and therefore should exist independent of hippocampus. Leyhe et al. (151) argue that information frequently retrieved in the past has to be consolidated by the hippocampus; once again, when this information had not been used for a time and thus the cortical network is weakened again. This may also apply to our findings of significant associations between hippocampal volume and remote semantic AM in patients and controls. Similarly, in functional imaging studies hippocampal activity (via PET, functional MRI) for remote autobiographical memories was more pronounced or more widespread than for recent ones (158–160).

Our results show that left in comparison to right hippocampus is significantly correlated with AM. A left-lateralized pattern of medial temporal lobe activation (via PET, functional MRI) was also frequently reported in functional imaging studies of AM (161, 162), which is explained by stimulus modality, with the left hippocampus being more involved in retrieval of contextual details of episodic memory and the right hippocampus in spatial memory (163–168). In addition, lesion studies show that damage to the left medial temporal lobe results more frequently in episodic memory impairment than right lateralized damage (169).

In the present study, mainly, the anterior hippocampus was significantly correlated with AM performance in the patient group [see also Ref. (170, 171)], while in the control group a specific topographical pattern of hippocampal involvement did not emerge. Similarly, significant correlations of anterior and posterior hippocampus with memory performance were reported in patients with schizophrenia and healthy subjects (15, 21). The assumption of a rostrocaudal distribution of hippocampal activity (via PET) during encoding and retrieval as described in the HIPER-model (23) was mitigated by findings of anterior and posterior hippocampal activation (via PET, functional MRI, event-related potentials by hippocampal electrodes) during both encoding and retrieval, respectively (172–174).

While interpreting the correlative results of AM in the present study, it should be noted that – in the face of the existing age variance within the patient group – the time interval relative to the lifetime periods examined varies in older and younger patients. Such influences can not be fully excluded as age during encoding and retrieval of information affect hippocampal involvement as does the oldness of the to-be-retrieved episode (175–179).

### Correlations between hippocampal volume and neuropsychology

According to our assumption among the additional neuropsychological parameters, we found verbal memory and working memory to be correlated primarily with right hippocampus. Executive functions, assessed by TMT B, showed significant negative associations with left and right hippocampal volume, indicating the smaller the hippocampus the more time is needed for alternating between numbers and letters. These associations only applied to the patient group.

A variety of studies investigating the relationship between neurocognition and hippocampal formation in schizophrenia has reported associations with verbal memory and executive functions (24, 25): Consistent with our results, positive correlations of hippocampal volume with parameters of verbal memory (assessed via Wechsler Memory Scale) were reported in patients with chronic schizophrenia and healthy subjects (88, 89). The present associations between right hippocampal volume and immediate and delayed verbal recall also correspond to previous findings (27, 89, 180). Sachdev et al. (27) found reduced verbal and visual memory performance and impaired executive functions in 20 elderly patients (64.4 ± 10.6 years of age) with chronic schizophrenia (duration of illness: 29.7 ± 16.6 years) in comparison to a somewhat older healthy control group (72.7 ± 6.7 years of age). Only in the patient group, right hippocampal volume was significantly correlated to verbal and visual memory. However, these results are frequently based on composite neuropsychological test scores or neuropsychological data reduced via factor analysis (27, 88, 89, 180, 181).

In the present study, executive impairments were significantly inversely correlated with left and right hippocampal volumes in the patient group. Likewise, in the above cited study by Sachdev et al. (27), frontal executive functions were significantly correlated with bilateral hippocampal volume, which applied only to the patient group. Executive functions, also measured, *interalia*, using the TMT B, were significantly correlated with anterior hippocampal volume in patients with first-episode (182) and chronic schizophrenia (14). These findings implicate that a possible neurodevelopmental hippocampal abnormality changes prefrontal structures or connections in adulthood (183–185), which may well explain the associations of hippocampus with executive functions (27, 182, 186).

In our study, working memory, assessed by the Digit Span backward, was significantly associated with bilateral hippocampal volume in the patient group. Similar results were reported in a VBM-study with impaired performance during the spatial span backwards being correlated with reduced gray matter volume of hippocampus (187). Structural and functional correlates (via functional MRI) of working memory were examined in a group of 154 individuals with schizophrenia, their siblings, and healthy controls. Results show positive correlations of hippocampal volume with working memory activity in dorsal anterior cingulate cortex and left inferior frontal gyrus, thus indicating long-distance structural–functional relationships (188).

To sum up, we found pronounced deficits of AM and additional neuropsychological parameters along with hippocampal volume reduction in a sample of older patients with chronic schizophrenia. While AM impairments seem to be relatively stable, other neuropsychological functions deteriorate cross-sectionally, which points at the complexity of AM. Our correlational findings extend previous results (99) and underline the relevance of volumetric hippocampal alterations in patients with chronic schizophrenia. However, a further examination of volumetric and neuropsychological changes in the course of the disease requires longitudinal data; therefore, a follow-up analysis is planned. Considering recent imaging findings of white matter abnormalities in schizophrenia potentially disconnecting the hippocampus from other structures [e. g., Ref. (185, 189)], further studies should also refer to the role of white matter in schizophrenia, using functional and structural imaging as well as neuropsychological investigations (190–192).

## Author Contributions

CH performed data collection, image processing, statistical analyses, and wrote the manuscript. ML and LS performed data collection, supported statistical analysis, and interpretation of data. ME supervised MRI procedures and clinical evaluation of the MRIs. PT, IF, and LK supported MRI data analysis and interpretation of data. US was involved in designing the study and interpretation of data. JS supervised study design and clinical assessments, supported data collection, and contributed to the interpretation of the results. All authors participated in critical revising and final approval of the manuscript, and agree to be accountable for all aspects of the work.

## Conflict of Interest Statement

The authors declare that the research was conducted in the absence of any commercial or financial relationships that could be construed as a potential conflict of interest.
